# MRI Study of Trauma-Induced Rotator Cuff Injuries and Their Correlation to Mechanism of Injury

**DOI:** 10.7759/cureus.113356

**Published:** 2026-07-25

**Authors:** Davidson Shandilya, Baidya Nath Singh, Vijendra Ruprela, Kashi Nath Sarkar, Amit Kumar, Saurabh Chaudhuri, Anoop Tiga, Roopshree Bode, Janganagari Anil, Rajshree Ganjeer

**Affiliations:** 1 Department of Radiodiagnosis, Raipur Institute of Medical Sciences, Raipur, IND; 2 Department of Orthopedics, Raipur Institute of Medical Sciences, Raipur, IND

**Keywords:** bone marrow edema, cofield classification, ellman grading, musculoskeletal radiology, rotator cuff tear, shoulder mri, shoulder trauma, supraspinatus tear, tendon retraction, trauma-induced injury

## Abstract

Introduction: Traumatic rotator cuff injury is a frequent cause of acute shoulder pain, weakness, and functional limitation. Unlike degenerative tears, trauma-related tears usually follow a definite injury event and may show specific imaging patterns depending on the mechanism of trauma. MRI allows detailed assessment of tendon integrity, tear morphology, retraction, muscle status, bone marrow edema, joint effusion, bursitis, and associated shoulder abnormalities. This study aimed to evaluate MRI findings in trauma-induced rotator cuff injuries and correlate them with the mechanism of injury.

Materials and methods: This prospective observational study included 50 patients with acute shoulder trauma who underwent MRI of the affected shoulder. Patients with predominant chronic degenerative rotator cuff disease or non-traumatic shoulder pathology were excluded. Clinical history regarding the mode of injury and onset of symptoms was recorded. Mechanisms of injury were categorized as fall on an outstretched hand, direct impact to the shoulder, traction injury, lifting or overhead loading injury, and high-energy trauma. MRI was performed using standard shoulder sequences in multiple planes. Tendon involvement, tear type, partial-thickness tear grade, full-thickness tear size, tendon retraction, acute injury markers, muscle changes, and associated intra-articular or periarticular findings were assessed. Ellman grading was used for partial-thickness tears, and Cofield classification was applied for full-thickness supraspinatus tears wherever applicable.

Results: The mean age of the study population was 47.98 ± 14.25 years. Male patients were more commonly affected, and right shoulder involvement was more frequent. The supraspinatus tendon was involved in all cases, either alone or as part of a multi-tendon injury pattern. Infraspinatus and subscapularis involvement were also identified, especially in more severe injuries. Partial-thickness tears were the most common tear pattern, while full-thickness tears and combined tear patterns were seen in selected cases. Tendon retraction, bone marrow edema, joint effusion, subacromial-subdeltoid bursitis, and acromioclavicular joint changes were observed in varying proportions. Mechanism of injury was significantly associated with tear type, full-thickness supraspinatus tear-size category, and bone marrow edema. High-energy trauma was predominantly associated with full-thickness tear components, larger supraspinatus tears, and bone marrow edema, whereas lower-energy mechanisms more commonly demonstrated partial-thickness tear patterns. No significant mechanism-based association was observed for Ellman grade, tendon retraction, or muscle edema.

Conclusion: MRI provides a comprehensive evaluation of trauma-induced rotator cuff injuries by characterizing tendon involvement, tear type and extent, tendon retraction, acute traumatic markers, and associated shoulder abnormalities. Correlation of MRI findings with the mechanism of injury may improve diagnostic confidence, assist in differentiating acute traumatic tears from chronic degenerative or acute-on-chronic pathology, and provide clinically relevant information for treatment planning.

## Introduction

Rotator cuff injuries are a major cause of shoulder pain, weakness, restricted movement, and functional limitation in adults. The rotator cuff is formed by the supraspinatus, infraspinatus, teres minor, and subscapularis tendons, which blend with the glenohumeral capsule and contribute to the dynamic stability of the shoulder joint [1]. The shoulder permits a wide range of motion, but this mobility depends on coordinated soft tissue support rather than deep bony constraint. During elevation and rotation, the rotator cuff compresses and centers the humeral head within the glenoid, counterbalancing the superior pull of the deltoid and maintaining smooth glenohumeral motion [2]. Disruption of this mechanism can produce pain, loss of strength, altered biomechanics, and difficulty with overhead activity.

Rotator cuff tears are common in clinical practice and may be traumatic, degenerative, or acute-on-chronic. Their prevalence increases with advancing age, and tears may also be detected in individuals without shoulder symptoms [3]. Population-based studies have similarly demonstrated that symptomatic and asymptomatic tears coexist, particularly in older adults [4,5]. Consequently, identification of a rotator cuff tear on imaging does not by itself establish that the lesion was caused by a recent traumatic event. Diagnosis of a trauma-induced rotator cuff injury, therefore, requires correlation of the clinical history, timing of symptom onset, mechanism of injury, and MRI findings.

Traumatic and chronic degenerative rotator cuff tears differ in their clinical presentation and imaging context. Degenerative tears generally develop gradually because of tendon attrition, repetitive microtrauma, age-related tendon changes, subacromial impingement, and reduced tendon quality. In contrast, traumatic tears typically follow a clearly identifiable injury and present with acute pain, weakness, restricted range of motion, or inability to elevate the arm. However, the terms “acute” and “traumatic” are not applied uniformly in the literature, which may affect both clinical decision-making and research interpretation [6]. A recently symptomatic tear may occur in a previously normal tendon or may represent an extension of pre-existing degenerative disease. Careful clinical and imaging correlation is therefore necessary to identify findings that support an acute traumatic component.

Several mechanisms may produce trauma-related rotator cuff injury. These include falling on an outstretched hand, direct impact to the shoulder, traction injury, lifting or overhead loading, shoulder dislocation, and high-energy trauma such as a road traffic accident. Each mechanism may transmit force differently across the shoulder girdle and may produce different patterns of tendon and soft tissue involvement. In patients above 40 years of age, rotator cuff tears may be detected after traumatic shoulder dislocation, and MRI is useful for identifying associated cuff injury in this setting [7]. High-energy trauma may produce larger tears, multi-tendon involvement, tendon retraction, bone marrow edema, labral injury, and capsuloligamentous injury. In contrast, lower-energy mechanisms may more commonly produce partial-thickness tears, focal tendon injury, bursitis, or localized marrow contusion.

The clinical relevance of mechanism-based assessment lies in its potential to improve diagnostic confidence and guide management. Acute traumatic tears, particularly in active patients, may require early orthopedic assessment because delay can allow tendon retraction, muscle atrophy, fatty infiltration, and reduction of tendon mobility. Acute tears of the rotator cuff have long been recognized as surgically important lesions, especially when they produce weakness and functional impairment [8]. In addition, traction or eccentric loading mechanisms may involve the musculotendinous junction, a pattern that can be specifically identified on MRI and may differ from the more common insertional supraspinatus tear [9]. A radiological report that describes only the presence of a tear may therefore be insufficient; the report should also describe the likely acuity, extent, tendon involvement, retraction, muscle status, and associated traumatic findings.

MRI is the preferred modality for the comprehensive evaluation of rotator cuff injuries. MRI provides excellent soft tissue contrast and allows multiplanar evaluation of tendons, muscles, bone marrow, cartilage, labrum, capsule, bursae, and adjacent soft tissues. Earlier work established the diagnostic value of MRI for detecting rotator cuff tears [10]. MRI is also useful for differentiating tendinosis, partial-thickness tear, full-thickness tear, complete tear, tendon retraction, muscle edema, fatty infiltration, joint effusion, subacromial-subdeltoid bursitis, and associated intra-articular lesions [11,12]. In the traumatic setting, MRI can demonstrate tendon discontinuity, surrounding edema, marrow contusion, acute bursal fluid, joint fluid, and associated soft tissue injury, all of which may help distinguish recent injury from chronic degenerative disease.

Anatomical and mechanical factors also influence the development and pattern of rotator cuff tears. Subacromial impingement has been described as an important contributor to chronic rotator cuff disease [13]. Acromial morphology, particularly a hooked acromion, has been associated with rotator cuff pathology and may predispose to chronic attritional tearing [14,15]. These factors are relevant in trauma-induced injuries because a patient may sustain an acute injury on a background of pre-existing structural predisposition. MRI can identify degenerative features such as acromioclavicular arthropathy, subacromial narrowing, tendon thinning, chronic tendinosis, superior humeral head migration, and muscle fatty change. Recognition of these findings helps differentiate purely traumatic tears from acute-on-chronic tears.

The supraspinatus tendon is the most commonly involved tendon in rotator cuff injury. Its location beneath the coracoacromial arch and its role in initiating shoulder abduction make it vulnerable to both degenerative and traumatic damage. Infraspinatus involvement is more often seen in larger posterosuperior tears, while subscapularis tears may occur with anterior shoulder trauma, forced external rotation, dislocation, or injury involving the biceps pulley mechanism [16]. Multi-tendon involvement is clinically significant because it may reflect a more severe injury and may be associated with greater weakness, instability, and need for surgical intervention. Therefore, a structured MRI assessment should specify whether the injury is isolated or involves multiple tendons.

Partial-thickness tears may involve the articular surface, bursal surface, or interstitial portion of the tendon. The Ellman classification remains a useful system for grading partial-thickness tears according to depth and helps communicate the severity of tendon involvement [17]. Full-thickness tears should be described by size, location, and degree of retraction. Accurate detection and quantification of rotator cuff tears are important because treatment decisions depend not only on whether a tear is present but also on its thickness, extent, and reparability [18]. Higher-field MRI and optimized shoulder protocols can further improve visualization of tendon fibers, tear margins, and associated intra-articular findings [19].

MRI findings may also provide clues regarding the timing of injury. Acute traumatic tears may show sharp tendon discontinuity, edema along the tendon or muscle, bone marrow edema at the greater tuberosity or humeral head, joint effusion, bursal fluid, and absence of advanced fatty atrophy. Chronic tears more commonly show tendon thinning, rounded margins, muscle atrophy, fatty infiltration, and superior migration of the humeral head. Recent studies have attempted to identify MRI features that help distinguish traumatic from degenerative tears, including specific signs related to tear configuration and surrounding tissue response [20]. Nevertheless, interpretation must remain clinically integrated, because traumatic symptoms may occur in patients with pre-existing asymptomatic tendon degeneration.

The natural history of rotator cuff disease further supports the need for accurate early evaluation. Asymptomatic tears may become symptomatic over time, and tear progression can occur, particularly when tendon quality is poor or mechanical stress persists [21]. In the context of trauma, identifying whether a tear is new, acute-on-chronic, or predominantly degenerative has practical implications for prognosis, counseling, and treatment planning. MRI-based characterization can help clinicians decide whether conservative treatment, physiotherapy, injection, arthroscopic debridement, or surgical repair is appropriate.

Despite extensive literature on rotator cuff imaging, relatively few studies have focused specifically on correlating the mechanism of trauma with MRI patterns of injury. Such correlation is valuable because patients with shoulder trauma may present with similar symptoms despite different injury mechanisms. A fall on an outstretched hand may produce an articular-sided supraspinatus injury; a direct blow may produce focal edema and bursal reaction; a traction injury may involve the musculotendinous junction; and high-energy trauma may produce larger tears with retraction and marrow edema. A mechanism-based imaging approach may therefore improve the clinical usefulness of MRI reports.

The present study aimed to evaluate the MRI features of trauma-induced rotator cuff injuries and determine their association with the mechanism of injury. The assessment included tendon involvement, tear type, partial-thickness tear grade, full-thickness tear size, tendon retraction, acute injury markers, muscle abnormalities, and associated shoulder findings. By integrating the clinical mechanism of injury with structured MRI evaluation, the study sought to provide a practical framework for the assessment of traumatic rotator cuff injuries and to improve communication between radiologists and treating clinicians.

## Materials and methods

This was a hospital-based prospective observational study conducted in the Department of Radiodiagnosis, Raipur Institute of Medical Sciences, Raipur, Chhattisgarh, India. The study was designed to evaluate MRI findings in patients with trauma-induced rotator cuff injuries and to correlate the imaging pattern with the documented mechanism of injury. No treatment, medication, surgical procedure, or interventional procedure was administered as part of the study. MRI was performed as part of routine clinical evaluation, and the research component was limited to a structured assessment of imaging findings and correlation with clinical history.

The study population included patients presenting with acute shoulder trauma and clinical suspicion of rotator cuff injury who were referred for MRI of the affected shoulder. A trauma-induced rotator cuff injury was defined as a rotator cuff abnormality occurring after a clearly identifiable traumatic event, followed by acute shoulder pain, weakness, or restriction of movement, with MRI findings supporting acute or subacute tendon injury. The mechanisms of injury were categorized as fall on an outstretched hand, direct impact to the shoulder, traction injury, lifting or overhead loading injury, and high-energy trauma such as a road traffic accident.

Patients were prospectively enrolled after Institutional Ethics Committee approval from August 14, 2024, to October 31, 2025. The sample size was predetermined as 50 patients based on recruitment feasibility, availability of eligible cases, and institutional patient load during the defined study period. No formal a priori sample-size or power calculation was performed. Eligible patients were consecutively enrolled until the predetermined sample size of 50 was achieved. Consecutive sampling was used to systematically recruit eligible patients and capture the clinical spectrum of trauma-related rotator cuff injuries encountered in routine radiology practice. Patients were included if they had a documented history of shoulder trauma, clinical suspicion of rotator cuff injury, MRI-confirmed rotator cuff abnormality, and consent for use of clinical and imaging data for research purposes. Patients were excluded if they had contraindications to MRI, pre-existing shoulder disease unrelated to trauma, inflammatory or infective shoulder pathology, predominant degenerative or atraumatic rotator cuff disease, or previous shoulder surgery likely to confound MRI interpretation.

Clinical data were recorded using a structured proforma before image analysis. The recorded variables included age, sex, affected side, mechanism of injury, time interval between trauma and MRI examination, clinical symptoms, and relevant examination findings. The mechanism of injury was treated as the primary independent variable. MRI-derived outcome variables included tendon involvement, type of tear, partial-thickness tear grade, full-thickness tear size, tendon retraction, acute traumatic markers, and associated intra-articular or periarticular abnormalities.

**Figure 1:**
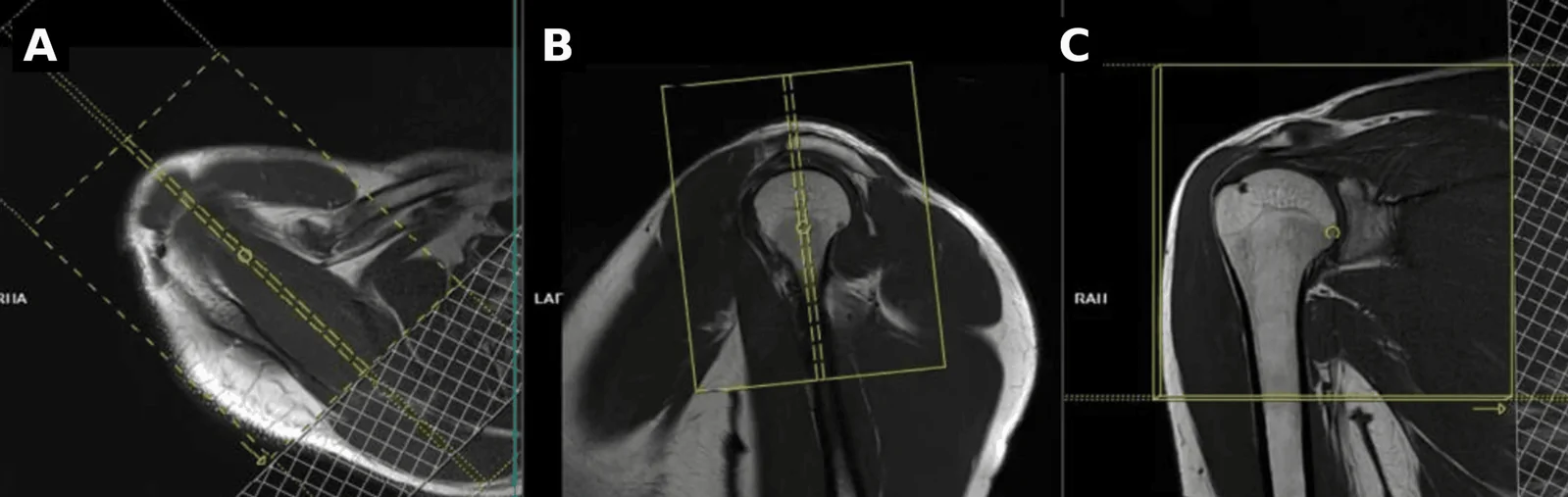
MRI shoulder protocol planning A: axial plane planning image used for evaluation of the subscapularis tendon, long head of biceps tendon, glenohumeral joint, labrum, and anterior/posterior periarticular structures; B: sagittal oblique plane planning image used for evaluation of rotator cuff muscles, muscle bulk, fatty infiltration, tendon orientation, and overall cuff morphology; and C: coronal oblique plane planning image used for evaluation of the supraspinatus and infraspinatus tendons, tendon footprint, subacromial-subdeltoid bursa, and acromioclavicular joint region.

MRI examinations were performed using a 1.5-T Siemens MAGNETOM Sempra MRI system (Siemens Healthineers AG, Erlangen, Germany) equipped with a dedicated shoulder surface coil. Patients were positioned supine, with the affected shoulder placed close to the magnet isocenter and the arm maintained in neutral or slight external rotation, as tolerated. Care was taken to minimize motion-related artifacts. The MRI shoulder protocol planning and acquisition planes are shown in Figure 1.

A standardized non-arthrographic shoulder MRI protocol was applied in all patients and included axial, coronal oblique, and sagittal oblique imaging. Axial proton density, fat-saturated, or T2 fat-saturated sequences were used to evaluate the subscapularis tendon, long head of the biceps tendon, labrum, joint effusion, and anterior and posterior periarticular structures. Coronal oblique fat-saturated sequences were used to assess the supraspinatus and infraspinatus tendons, tendon footprint, bursal surface, and subacromial-subdeltoid bursa. Sagittal oblique T1-weighted and fluid-sensitive sequences were used to evaluate overall cuff morphology, muscle bulk, atrophy, and fatty infiltration. Short tau inversion recovery (STIR) imaging was used where required for assessment of soft-tissue and bone marrow edema. Images were acquired with a slice thickness of 3 mm, an interslice gap of 0.3 mm, a field of view of 16 cm, and an acquisition matrix of 320 × 256. Sequence-specific repetition and echo times were selected according to the institutional shoulder MRI protocol. MR arthrography and abduction and external rotation (ABER)-position imaging were not performed.

MRI examinations were interpreted in a structured manner by two radiologists, each with eight years of experience in musculoskeletal MRI interpretation. Because the study specifically aimed to correlate MRI findings with the documented mechanism of injury, both radiologists were aware of the clinical history and mechanism of trauma at the time of image interpretation. The supraspinatus, infraspinatus, teres minor, and subscapularis tendons were evaluated for integrity, signal abnormality, thickening or thinning, tear morphology, tear depth, tear size, tendon retraction, and associated muscle changes. Partial-thickness tears were defined as incomplete tendon fiber disruption and were assessed according to the involved surface and tear depth, whereas full-thickness tears were defined as complete tendon discontinuity extending from the articular to the bursal surface. Interobserver agreement was not formally assessed.

Standard classification systems were applied wherever appropriate. Partial-thickness tears were graded according to the Ellman classification, which categorizes incomplete rotator cuff tears based on depth of tendon involvement [17]. Full-thickness supraspinatus tear components were categorized according to tear size using the Cofield classification [8]. Tendon retraction was recorded as present or absent. Muscle atrophy and fatty infiltration were also documented. Acute MRI injury markers included tendon edema, muscle edema, bone marrow edema, joint effusion, soft tissue edema, and subacromial-subdeltoid bursitis. Associated findings such as long head of biceps tendon abnormality, labral abnormality, acromioclavicular joint changes, fracture, and other traumatic periarticular findings were also recorded.

The primary outcome variables were tendon involvement, type of tear, partial-thickness tear grade, full-thickness tear size, tendon retraction, and acute MRI injury markers. Secondary variables included associated shoulder abnormalities such as bursitis, joint effusion, biceps tendon pathology, labral abnormality, fracture, and bone marrow edema. The main analysis focused on the association between the mechanism of injury and MRI-defined rotator cuff injury pattern.

Data were entered into Microsoft Excel (Microsoft Corporation, Redmond, Washington, United States) and analyzed using IBM SPSS Statistics for Windows, Version 23 (Released 2015; IBM Corp., Armonk, New York, United States). Continuous variables were expressed as mean ± standard deviation, median, and range where appropriate. Categorical variables were expressed as frequencies and percentages. The association between mechanism of injury and MRI findings was assessed using the chi-square test or Fisher’s exact test, depending on expected cell counts. Student’s t-test or analysis of variance was used for continuous variables where applicable. A p-value of less than 0.05 was considered statistically significant. No multivariable regression analysis was performed because of the modest sample size and small subgroup counts across some mechanism-based categories.

The study was approved by the Institutional Ethics Committee of Raipur Institute of Medical Sciences, Raipur, Chhattisgarh, India (IEC/RIMS/2024/091; August 14, 2024). Written informed consent was obtained from all participants before enrollment. Patient confidentiality was maintained, and no personal identifiers were included in the data analysis, manuscript, or imaging figures. All MRI examinations were performed as part of routine clinical care, and no additional imaging, treatment, or intervention was undertaken solely for research purposes.

## Results

A total of 50 patients with trauma-induced rotator cuff injury were included in the study. The mean age of the study population was 47.98 ± 14.25 years. Most patients belonged to the 30-50-year age group, followed closely by those above 50 years. The age distribution is summarized in Table 1. A male predominance was observed, with 36 males and 14 females. Right shoulder involvement was more common than left shoulder involvement, with the right side affected in 34 patients. The gender and side distribution are summarized in Table 1.

**Table 1:** Demographic and clinical profile of the study population This table summarizes the demographic and clinical characteristics of the study population, including age distribution, mean age, age range, gender distribution, side of shoulder involvement, and total number of patients included in the study.

Variable	Category / Statistic	Frequency / Value
Age	<30 years	5
	30–50 years	23
	>50 years	22
	Mean ± SD	47.98 ± 14.25 years
	Range	22–70 years
Gender	Male	36
	Female	14
Side of involvement	Right	34
	Left	16
Total	Study population	50

**Figure 2:**
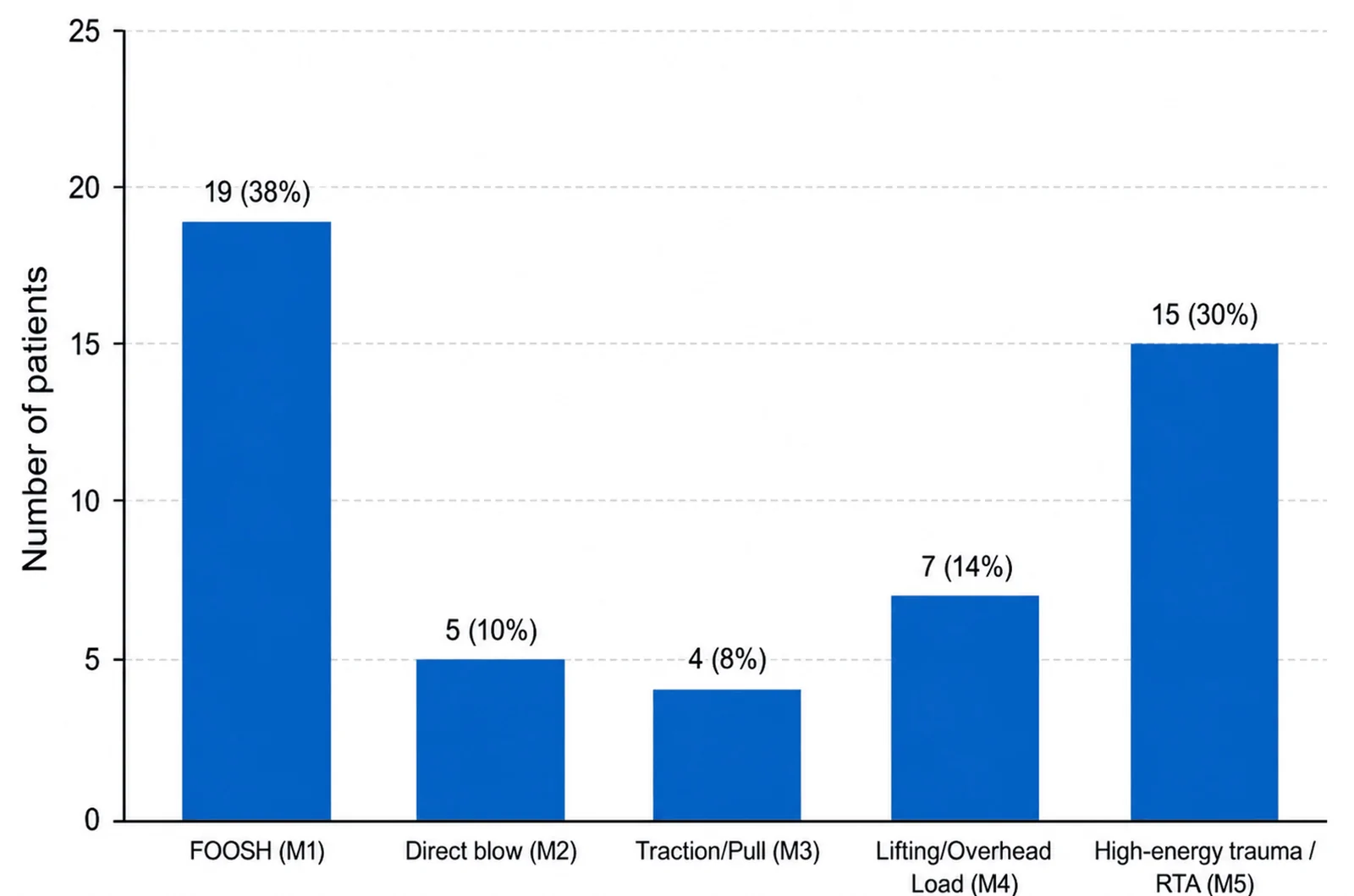
Mechanism of injury distribution Bar chart showing the distribution of mechanisms of injury among 50 patients with trauma-induced rotator cuff injuries. Fall on an outstretched hand (FOOSH) was the most common mechanism, followed by high-energy trauma/road traffic accident. Values are shown as the number of patients with corresponding percentages.

Fall on an outstretched hand was the most common mechanism of injury and was observed in 19 patients. High-energy trauma was the second most frequent mechanism and was observed in 15 patients. Lifting or overhead loading injury was observed in seven patients, direct impact to the shoulder in five patients, and traction injury in four patients. The mechanism of injury distribution is shown in Figure 2.

**Figure 3:**
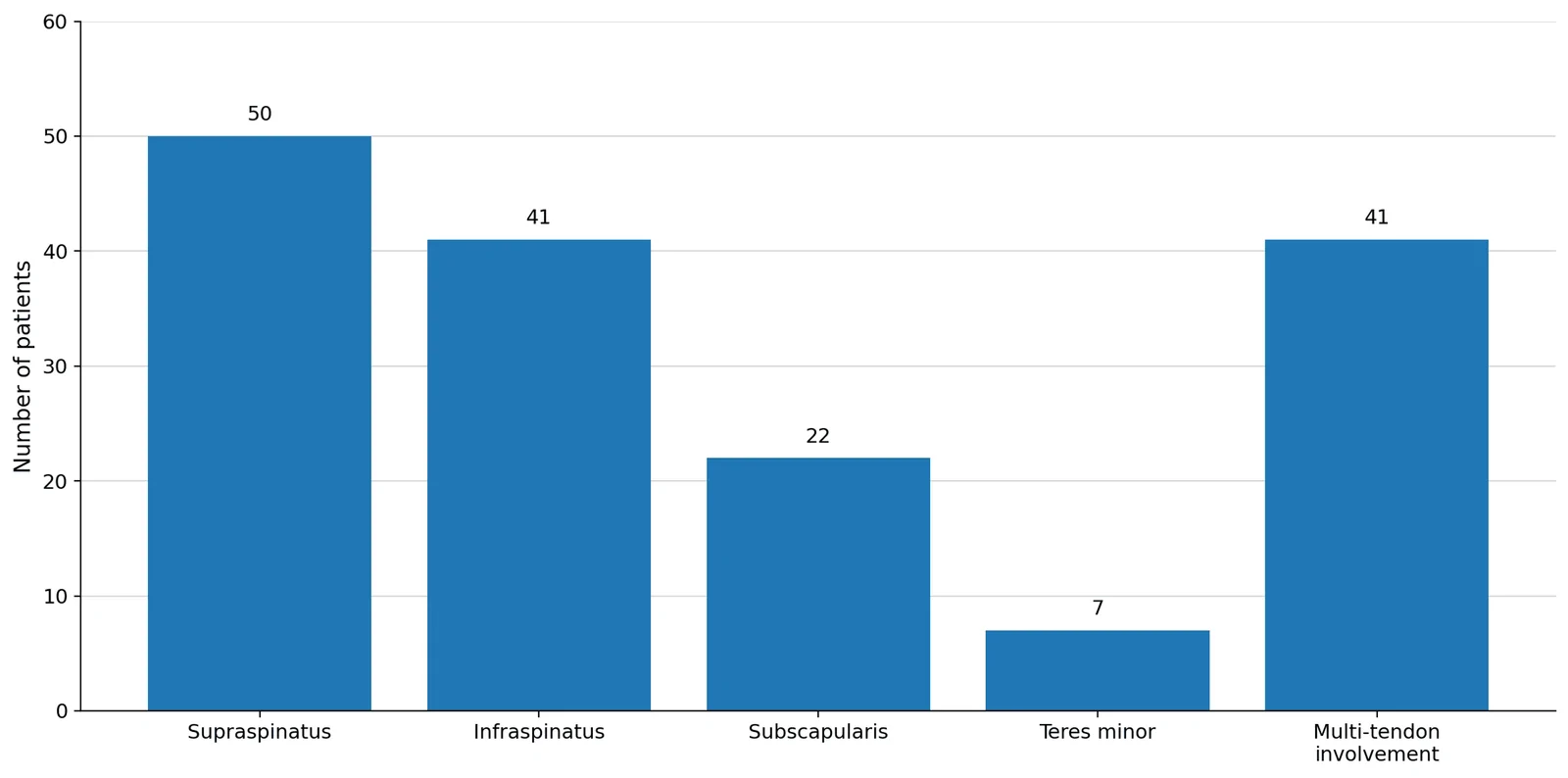
Tendon involvement pattern Bar chart showing the distribution of rotator cuff tendon involvement among patients with trauma-induced rotator cuff injuries. The Y-axis represents the number of patients. Supraspinatus involvement was present in 50 patients, followed by infraspinatus involvement in 41 patients, subscapularis involvement in 22 patients, teres minor involvement in seven patients, and multi-tendon involvement in 41 patients.

MRI demonstrated that the supraspinatus tendon was involved in all patients. Infraspinatus involvement was seen in 41 patients, while subscapularis involvement was noted in 22 patients. Teres minor involvement was observed in seven patients, but was not identified as a dominant, isolated injury pattern. Multi-tendon involvement was common and was observed in 41 patients, indicating that trauma-induced rotator cuff injuries frequently extend beyond the supraspinatus tendon alone. The tendon involvement pattern is shown in Figure 3 and summarized in Table 2.

**Table 2:** Tendon involvement pattern This table summarizes the distribution of rotator cuff tendon involvement in the study population. Supraspinatus involvement was observed in all patients, followed by infraspinatus, subscapularis, and teres minor involvement. Multi-tendon injury patterns were frequent.

Variable	Tendon involved	Frequency	Percentage
Tendon involvement	Supraspinatus	50	100%
	Infraspinatus	41	82%
	Subscapularis	22	44%
	Teres minor	7	14%
	Multi-tendon involvement	41	82%

Partial-thickness tears were the most frequent pattern of rotator cuff injury. Isolated partial-thickness tears were seen in 25 patients, while combined partial-thickness and full-thickness tear components were seen in 22 patients. No patient showed an isolated full-thickness tear without an associated partial-thickness component. A small number of patients showed tendinous injury without a clearly measurable full-thickness defect. The distribution of tear types is summarized in Table 3.

**Table 3:** Type of rotator cuff tear This table summarizes the distribution of rotator cuff tear patterns in the study population. Partial-thickness tears were the most common finding, followed by combined partial- and full-thickness tear components. No isolated full-thickness tear was observed.

Variable	Category	Frequency	Percentage
Type of rotator cuff tear	Partial only	25	50%
	Full only	0	0%
	Both (Partial + Full)	22	44%
	Tendinosis only	3	6%
	Total	50	100%

Among partial-thickness supraspinatus tears, Ellman grading was applied according to tear depth. Most partial-thickness tears were distributed across low- to intermediate-grade categories, with higher-grade partial tears seen in selected cases. The distribution of partial-thickness supraspinatus tears according to Ellman grading is presented in Table 4.

**Table 4:** Ellman grading of partial-thickness supraspinatus tears This table shows the distribution of partial-thickness supraspinatus tears according to Ellman grading. Grade I tears were the most frequent, followed by Grade II and Grade III tears.

Grade	Frequency	Percentage
Grade I	10	40%
Grade II	9	36%
Grade III	6	24%
Total	25	100%

Full-thickness supraspinatus tear components were categorized according to maximum tear dimension using the Cofield classification. This classification helped separate full-thickness tears into clinically meaningful size groups and allowed comparison of tear burden across different mechanisms of injury. The distribution is presented in Table 5.

**Table 5:** Full-thickness supraspinatus tear size by Cofield classification Full-thickness supraspinatus tear components were categorized according to the Cofield classification. Large tears represented the predominant full-thickness tear pattern, while medium tears were uncommon, and no small or massive tears were observed.

Variable	Cofield category	Frequency	Percentage
Full-thickness supraspinatus tear size	Small	0	0%
	Medium	1	4.5%
	Large	21	95.5%
	Massive	0	0%
	Total	22	100%

Tendon retraction was evaluated as an indicator of tear severity and potential repair complexity. Retraction was identified in selected cases, particularly where the tear morphology was more extensive; however, most patients did not show measurable retraction. The distribution of tendon retraction is presented in Table 6.

**Table 6:** Tendon retraction distribution Tendon retraction was assessed on MRI in all patients. Retraction was present in a minority of cases, while most patients showed no measurable tendon retraction, suggesting that advanced retraction was not a dominant feature in the study population.

Variable	Retraction status	Frequency	Percentage
Tendon retraction	Present	8	16%
	Absent	42	84%
	Total	50	100%

Acute MRI injury markers were assessed to identify imaging features supporting recent traumatic injury. Tendon edema was the most consistent marker, followed by muscle edema. Bone marrow edema was less frequent but provided additional evidence of osseous impact or contusion in selected cases. The distribution of acute MRI injury markers is presented in Table 7.

**Table 7:** Acute MRI injury markers Acute MRI injury markers were recorded to support the traumatic or subacute nature of rotator cuff injury. Tendon edema was present in all patients, followed by muscle edema, while bone marrow edema was observed in less than half of the study population.

Variable	MRI finding	Frequency	Percentage
Acute MRI injury markers	Tendon edema	50	100%
	Muscle edema	47	94%
	Bone marrow edema (contusion)	22	44%

Associated MRI findings were reviewed to determine the additional intra-articular and periarticular abnormalities accompanying traumatic rotator cuff injury. Joint effusion was the most consistent associated finding, while subacromial-subdeltoid bursitis, long head of biceps tendon pathology, fracture, and labral tear were observed in varying proportions. The distribution of associated MRI findings is presented in Table 8.

**Table 8:** Associated MRI findings Associated MRI findings were recorded to assess the extent of traumatic shoulder involvement beyond the rotator cuff tendons. Joint effusion was present in all patients, followed by subacromial-subdeltoid (SASD) bursitis, long head of biceps tendon (LHBT) pathology, fracture, and labral tear.

Variable	Finding	Frequency	Percentage
Associated MRI findings	Joint effusion	50	100%
	SASD bursitis	38	76%
	LHBT pathology	30	60%
	Fracture	29	58%
	Labral tear	15	30%

Tear pattern varied according to the mechanism of injury. All 15 patients with high-energy trauma demonstrated a full-thickness tear component, whereas direct blow, traction, and lifting or overhead loading injuries were associated exclusively with partial-thickness tears. Fall on an outstretched hand showed a mixed pattern, comprising nine partial-thickness tears and seven full-thickness tear components. These findings are summarized in Table 9.

**Table 9:** Association between mechanism of injury and type of tear This table shows the distribution of partial-thickness and full-thickness rotator cuff tear components according to the mechanism of injury. Tendinosis-only cases were not included in this tear-type comparison; therefore, the total number analyzed in this table is 47. FOOSH: fall on an outstretched hand; RTA: road traffic accident

Variable	Mechanism of injury	Partial-thickness tear	Full-thickness component	Total
Mechanism vs type of tear	FOOSH (M1)	9	7	16
	Direct blow (M2)	5	0	5
	Traction/Pull (M3)	4	0	4
	Lifting/Overhead Load (M4)	7	0	7
	High-energy trauma/RTA (M5)	0	15	15
	Total	25	22	47

The association between the mechanism of injury and the type of rotator cuff tear was tested separately using the chi-square test. A statistically significant association was observed, indicating that the tear pattern varied according to the mechanism of injury. The statistical analysis is presented in Table 10.

**Table 10:** Statistical analysis of mechanism of injury versus type of tear The chi-square test demonstrated a statistically significant association between the mechanism of injury and the type of rotator cuff tear. High-energy trauma was predominantly associated with full-thickness tear components.

Analysis	Test used	χ² value	df	p-value	Statistical inference	Interpretation
Mechanism of injury vs type of tear	Chi-square test	31.19	4	<0.001	Statistically significant	Mechanism of injury showed a significant association with tear type. High-energy trauma was predominantly associated with full-thickness components, while direct blow, traction, and lifting/overhead mechanisms showed partial-thickness tears.

Full-thickness supraspinatus tear size was compared across different mechanisms of injury using the Cofield classification. Fall on an outstretched hand injuries showed both medium and large tear components, whereas high-energy trauma was associated with large full-thickness tear components. The distribution of Cofield tear-size categories according to the mechanism of injury is presented in Table 11.

**Table 11:** Association between mechanism of injury and Cofield classification This table shows the distribution of full-thickness supraspinatus tear-size categories according to mechanism of injury using the Cofield classification. FOOSH: fall on an outstretched hand; RTA: road traffic accident

Variable	Mechanism of injury	Small	Medium	Large	Massive	Total
Mechanism vs Cofield classification	FOOSH (M1)	0	1	6	0	7
	Direct blow (M2)	0	0	0	0	0
	Traction/Pull (M3)	0	0	0	0	0
	Lifting/Overhead Load (M4)	0	0	0	0	0
	High-energy trauma/RTA (M5)	0	0	15	0	15
	Total	0	1	21	0	22

The relationship between the mechanism of injury and full-thickness supraspinatus tear size was tested separately. Because multiple cells had zero values, Fisher’s exact test was used for statistical analysis. A statistically significant association was observed, indicating that larger full-thickness tear components were concentrated in high-energy trauma. The statistical analysis is presented in Table 12.

**Table 12:** Statistical analysis of mechanism of injury versus Cofield classification Fisher’s exact test demonstrated a statistically significant association between the mechanism of injury and full-thickness supraspinatus tear size. Large tears were predominantly observed in high-energy trauma.

Analysis	Test used	Test statistic	df	p-value	Statistical inference	Interpretation
Mechanism of injury vs Cofield classification	Fisher’s exact test	—	—	<0.001	Statistically significant	Full-thickness supraspinatus tear size showed a significant association with the mechanism of injury, with large tears predominantly observed in high-energy trauma.

Acute MRI injury markers were compared across different mechanisms of injury. Tendon edema was present in all cases, while muscle edema was also frequently observed across most mechanisms. Bone marrow edema showed a more mechanism-specific pattern, with higher occurrence in high-energy trauma and direct-blow injuries. The distribution of acute MRI injury markers according to mechanism of injury is presented in Table 13.

**Table 13:** Association between mechanism of injury and acute MRI injury markers This table shows the distribution of acute MRI injury markers across different mechanisms of injury. Tendon edema was present in all groups, while bone marrow edema was concentrated in high-energy trauma and direct blow injuries. FOOSH: fall on an outstretched hand; RTA: road traffic accident

Variable	Mechanism of injury	Muscle edema	Tendon edema	Bone marrow edema	Total patients
Mechanism vs acute MRI injury markers	FOOSH (M1)	16	19	2	19
	Direct blow (M2)	5	5	5	5
	Traction/Pull (M3)	4	4	0	4
	Lifting/Overhead Load (M4)	7	7	0	7
	High-energy trauma/RTA (M5)	15	15	15	15
	Total positive findings	47	50	22	50

The relationship between the mechanism of injury and acute MRI injury markers was tested separately. Tendon edema was not suitable for statistical comparison because it was present in all mechanism groups. Muscle edema did not show a statistically significant association with the mechanism of injury. Bone marrow edema showed a statistically significant association, indicating that osseous contusion was more strongly related to high-impact mechanisms. The statistical analysis is presented in Table 14.

**Table 14:** Statistical analysis of mechanism of injury versus acute MRI injury markers Bone marrow edema showed a statistically significant association with the mechanism of injury, while muscle edema did not show a significant mechanism-based difference. Tendon edema was not tested because it was present in all patients.

Analysis	MRI marker	Test used	χ² value	df	p-value	Statistical inference	Interpretation
Mechanism of injury vs acute MRI injury markers	Tendon edema	Not applicable	—	—	—	Not applicable	Tendon edema was present in all mechanism groups, so comparative testing was not meaningful.
	Muscle edema	Chi-square test	5.21	4	0.267	Not statistically significant	Muscle edema was common across mechanisms and did not show a significant mechanism-based difference.
	Bone marrow edema	Chi-square test	42.74	4	<0.001	Statistically significant	Bone marrow edema showed a strong association with the mechanism of injury and was concentrated in high-energy trauma and direct blow.

No statistically significant association was found between the mechanism of injury and Ellman grade among partial-thickness tears. Similarly, tendon retraction did not show a statistically significant association with the mechanism of injury. Tendon involvement was not included in formal statistical testing because supraspinatus involvement was present in all cases, limiting meaningful comparative analysis. The overall statistical significance summary for key mechanism-based MRI correlations is presented in Table 15.

**Table 15:** Summary of mechanism-based statistical associations This table summarizes the statistical significance of key mechanism-based MRI correlations. Significant associations were observed for tear type, Cofield tear-size category, and bone marrow edema, while Ellman grade, tendon retraction, and muscle edema did not show significant mechanism-based associations.

Variable compared	Test used	p-value	Significant	Interpretation
Mechanism vs type of tear	Chi-square test	<0.001	Yes	Tear pattern varied significantly with the mechanism of injury.
Mechanism vs Ellman grade	Fisher’s exact test	>0.05	No	Partial-thickness tear grade did not differ significantly by mechanism.
Mechanism vs Cofield classification	Fisher’s exact test	<0.001	Yes	Full-thickness tear size showed significant mechanism-based variation.
Mechanism vs tendon retraction	Chi-square test	>0.05	No	Tendon retraction did not show a significant mechanism-based association.
Mechanism vs bone marrow edema	Chi-square test	<0.001	Yes	Bone marrow edema was significantly associated with high-impact mechanisms.
Mechanism vs muscle edema	Chi-square test	>0.05	No	Muscle edema did not show a significant mechanism-based difference.
Mechanism vs tendon involvement	Not applied	—	—	Formal testing was not performed because supraspinatus involvement was present in all cases.
Mechanism vs tendon edema	Not applicable	—	—	Formal testing was not applicable because tendon edema was present in all patients.

**Figure 4:**
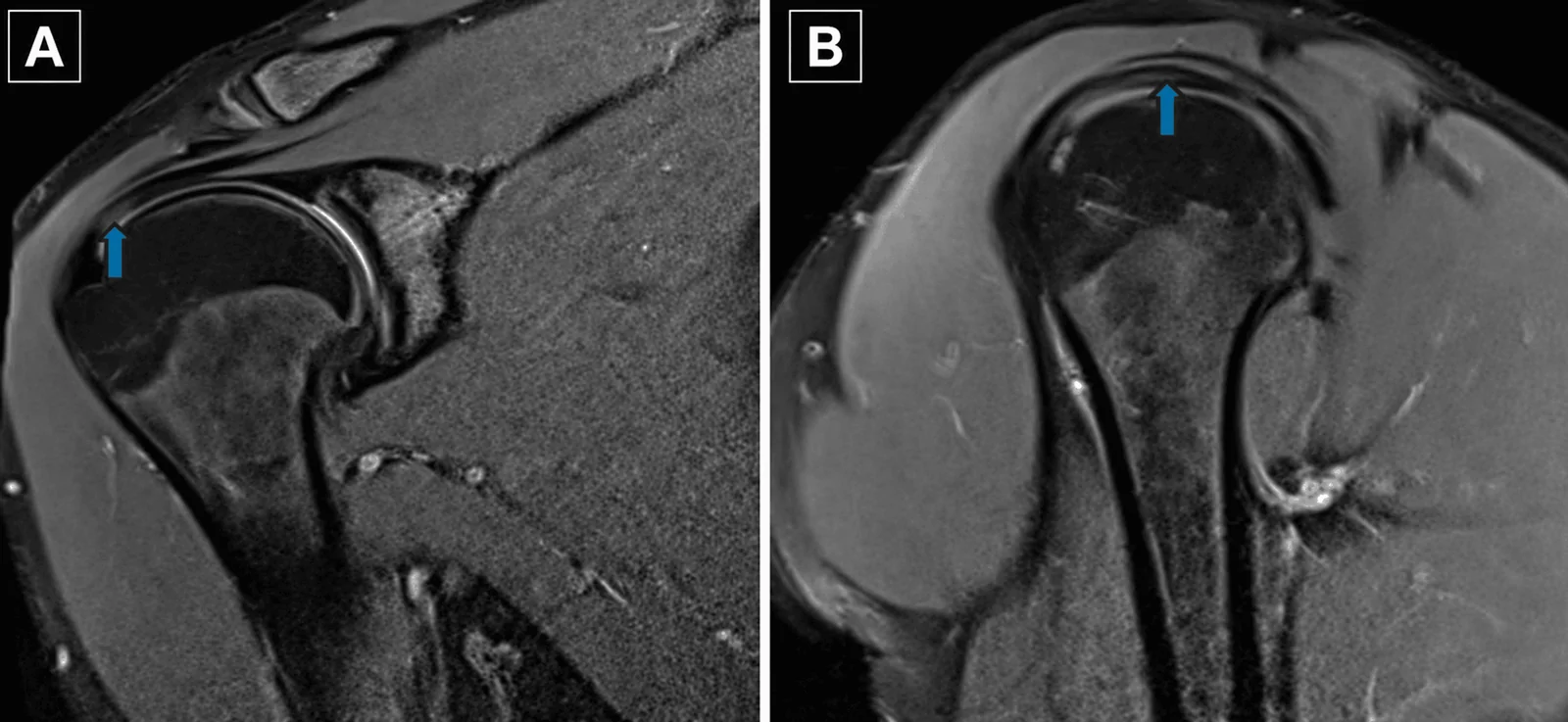
Supraspinatus tendinosis on coronal and sagittal MRI A: coronal proton density fat-saturated MRI image demonstrates a patchy intratendinous hyperintense signal within the supraspinatus tendon without definite extension to the articular or bursal surface (arrow); B: sagittal proton density fat-saturated MRI image shows preserved tendon fiber continuity and alignment, consistent with supraspinatus tendinosis (arrow).

**Figure 5:**
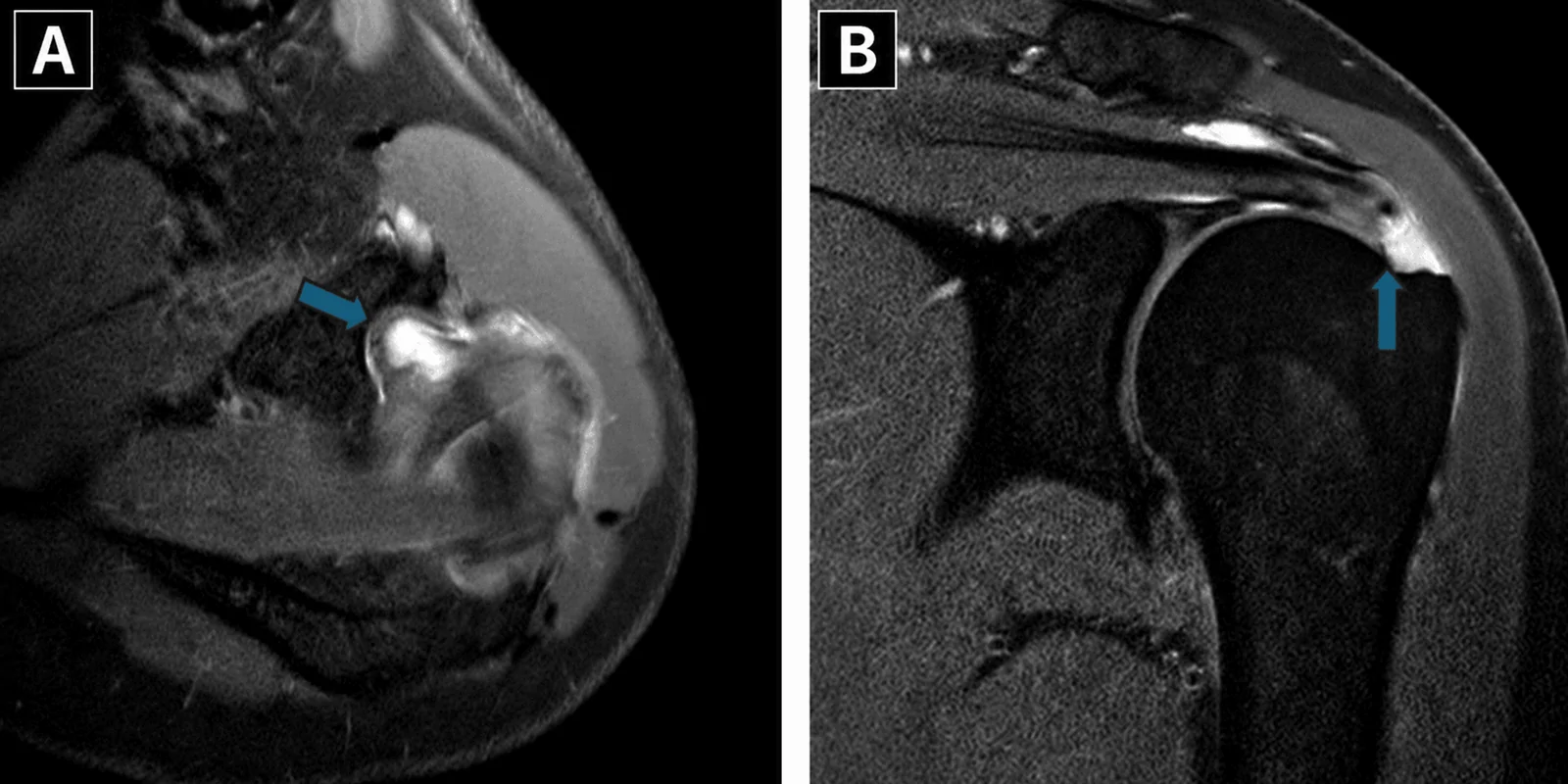
Full-thickness U-shaped supraspinatus tendon tear on MRI A: axial proton density fat-saturated MRI image demonstrates a full-thickness tear involving the distal supraspinatus tendon, with fluid signal extending through the tendon defect; B: coronal proton density fat-saturated MRI image shows transmural discontinuity of the supraspinatus tendon fibers at the footprint, with fluid signal extending from the articular surface to the bursal surface and associated subacromial-subdeltoid bursal fluid. The arrows indicate the site of tendon disruption.

**Figure 6:**
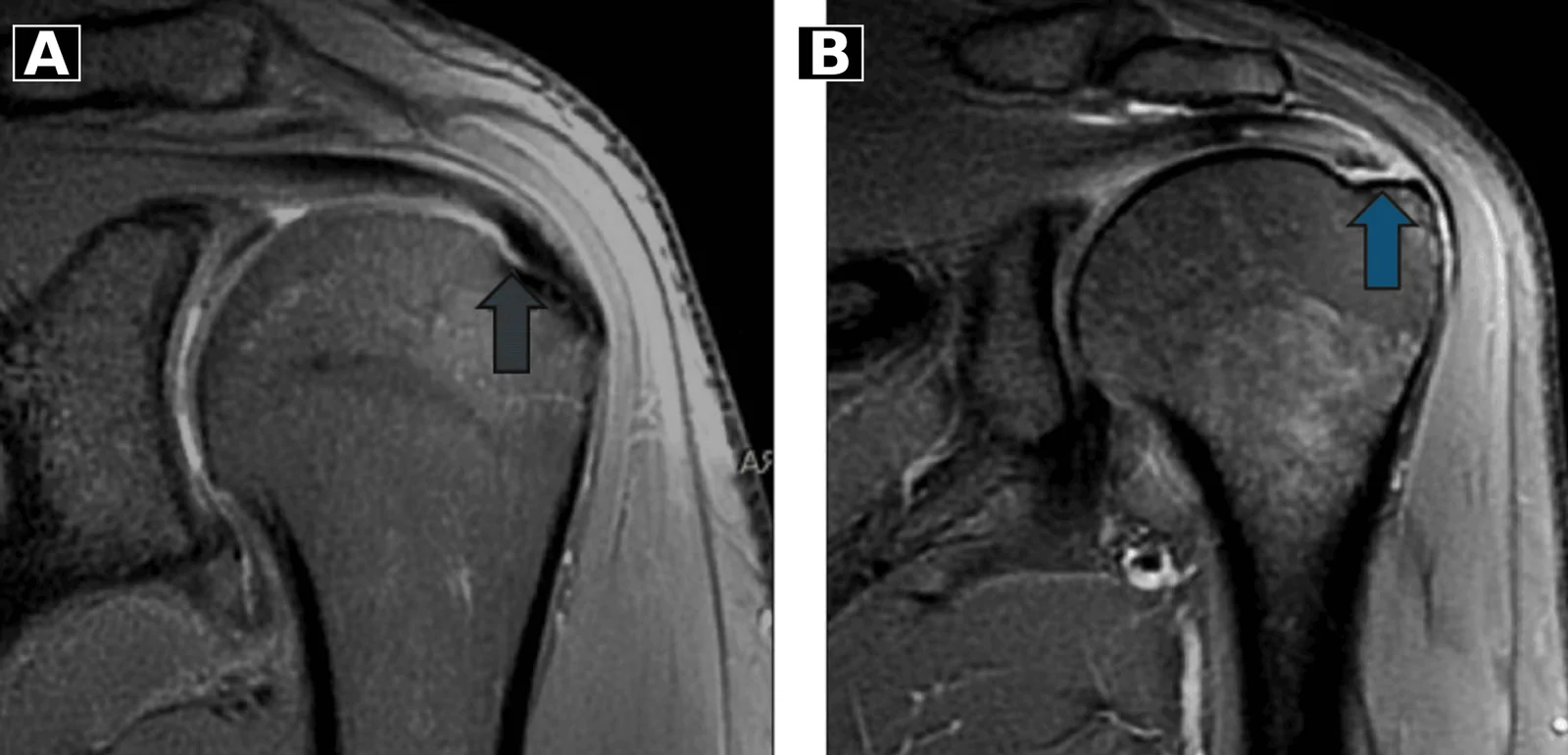
Ellman Grade I and Grade III partial-thickness supraspinatus tears on MRI A: coronal proton density fat-saturated MRI image demonstrates a low-grade partial-thickness tear of the supraspinatus tendon at the footprint, consistent with an Ellman Grade I tear (the arrow indicates focal superficial tendon fiber disruption involving less than 3 mm depth); B: coronal proton density fat-saturated MRI image demonstrates a high-grade partial-thickness supraspinatus tendon tear with deep fluid-signal extension at the footprint, consistent with an Ellman Grade III tear (the arrow indicates tendon fiber disruption involving more than 6 mm depth and more than 50% of tendon thickness).

Representative MRI images are shown in Figures 4-6. Figure 4 demonstrates supraspinatus tendinosis on coronal and sagittal proton density fat-saturated MRI images. Figure 5 demonstrates a full-thickness U-shaped supraspinatus tendon tear on axial and coronal proton density fat-saturated MRI images. Figure 6 demonstrates Ellman Grade I and Grade III partial-thickness supraspinatus tears on coronal proton density fat-saturated MRI images.

These images illustrate the role of multiplanar fat-saturated MRI sequences in identifying tendon signal abnormality, tendon discontinuity, tear depth, tear extent, retraction, and associated traumatic changes.

Overall, MRI enabled systematic characterization of trauma-induced rotator cuff injuries according to tendon involvement, tear type, tear grade, tear size, tendon retraction, acute injury markers, and associated shoulder findings. Supraspinatus involvement was observed in all patients, and multi-tendon involvement was frequent. Partial-thickness tears were the predominant pattern, either alone or in combination with full-thickness tear components. Mechanism-based analysis showed significant associations with tear type, full-thickness supraspinatus tear-size category, and bone marrow edema, with high-energy trauma predominantly associated with full-thickness tear components, larger tears, and osseous contusion.

## Discussion

The present study evaluated MRI patterns of trauma-induced rotator cuff injuries and correlated them with the mechanism of injury. The principal findings were that supraspinatus involvement was present in all patients, multi-tendon involvement was frequent, partial-thickness tears were the dominant tear pattern, and high-energy trauma was associated with more severe tear morphology, larger full-thickness tear components, and bone marrow edema. These findings support a mechanism-based approach to traumatic shoulder MRI, in which the radiologist interprets tendon abnormalities in relation to the clinical event rather than reporting the tear as an isolated structural finding.

The universal involvement of the supraspinatus tendon in this study is anatomically and biomechanically plausible. The supraspinatus tendon occupies a vulnerable position beneath the coracoacromial arch and plays an important role in initiating abduction and stabilizing the humeral head during motion [1,2]. Because it is exposed to tensile loading, compression, and shear forces during traumatic shoulder events, it is often the first or most consistently involved tendon. The high frequency of associated infraspinatus and subscapularis involvement in this study suggests that many traumatic cuff injuries are not limited to the supraspinatus footprint alone but may reflect wider force transmission across the cuff complex.

A major diagnostic challenge in traumatic shoulder imaging is distinguishing recent traumatic tearing from pre-existing degeneration. Rotator cuff tears are increasingly common with age and may be found in asymptomatic individuals [3-5]. Therefore, in a patient presenting after trauma, the presence of a tear should be interpreted with caution unless supported by clinical timing and acute MRI markers. In the present study, tendon edema was seen in all cases, muscle edema was common, and bone marrow edema showed a mechanism-based distribution. These findings support the view that traumatic cuff assessment should include not only tendon discontinuity but also surrounding soft tissue and osseous response.

The need for clear terminology is important. The literature has shown variability in how the terms “acute” and “traumatic” are defined [6]. Some authors emphasize symptom duration, while others focus on a definite injury event or imaging features suggesting recent tissue damage. The present study used a clinical-radiological approach, including patients with a clear traumatic event and MRI findings supporting acute or subacute injury. This approach is relevant in daily practice because many patients may have both recent trauma and background tendon degeneration.

Mechanism of injury showed a meaningful relationship with MRI pattern. Fall on an outstretched hand showed a mixed distribution of partial-thickness and full-thickness components. Direct blow showed a closer association with osseous and periarticular traumatic changes, particularly bone marrow edema. Traction and lifting or overhead loading mechanisms were more often associated with partial-thickness patterns. High-energy trauma showed the most severe spectrum, with a greater frequency of full-thickness components, larger tear size, and bone marrow edema. Similar mechanism-related variation has been reported in traumatic shoulder settings, including rotator cuff tears following shoulder dislocation in older patients [7]. These observations support incorporating the mechanism of injury into MRI interpretation whenever clinical information is available.

Full-thickness supraspinatus tear components were evaluated using the Cofield classification, and larger tears were predominantly observed in high-energy trauma. This is clinically important because tear size influences repair planning, surgical difficulty, and prognosis. Acute full-thickness tears have long been recognized as important traumatic lesions, particularly when associated with functional weakness [8]. In the present study, high-energy trauma was the mechanism most strongly associated with large tear components, which is consistent with the greater force transmission expected in road traffic accidents or similar high-impact mechanisms.

The evaluation of the musculotendinous junction is also relevant in traumatic shoulder MRI. Traction and eccentric loading may produce injury away from the tendon footprint, and MRI can identify edema or disruption at the myotendinous junction [9]. Although the present study primarily categorized tendon involvement, tear type, tear size, and acute injury markers, the frequent presence of muscle edema reinforces the importance of examining the entire musculotendinous unit. A narrow focus on the distal tendon insertion may underestimate the extent of traumatic injury.

MRI was central to this study because it allowed systematic assessment of the cuff tendons, tear morphology, tear depth, tendon retraction, muscle edema, marrow edema, joint effusion, bursitis, labral abnormality, biceps tendon pathology, and fracture-related findings. Earlier MRI studies established the diagnostic value of MRI in detecting rotator cuff tears [10], and subsequent reviews have emphasized the broader role of MRI in evaluating external impingement, tendon degeneration, and associated shoulder abnormalities [11,12]. In the present study, fat-saturated proton density sequences were particularly useful for demonstrating tendon signal abnormality, fluid signal traversing a tear, peritendinous edema, and marrow contusion.

The study also confirms that anatomical and degenerative factors remain important even in traumatic cases. Subacromial impingement and acromial morphology are recognized contributors to rotator cuff pathology [13-15]. In a patient with trauma, such background factors may influence whether a tendon tears, the extent of tearing, and whether the injury represents a purely traumatic lesion or an acute-on-chronic event. This distinction is clinically relevant because traumatic tears in healthier tendons may behave differently from tears occurring in chronically degenerated tissue.

Subscapularis assessment deserves specific attention. Subscapularis tears may be associated with anterior shoulder trauma, dislocation, forced external rotation, or biceps pulley injury [16]. In this study, subscapularis involvement was less frequent than supraspinatus and infraspinatus involvement but remained clinically important when present. In routine reporting, subscapularis and biceps pulley evaluation should not be overlooked, especially in patients with anterior shoulder symptoms or traumatic instability.

Partial-thickness tears were the dominant tear pattern in this cohort. Ellman grading allowed practical classification of these tears according to depth [17]. The absence of a significant relationship between the mechanism of injury and Ellman grade suggests that partial tear depth may depend on multiple factors, including tendon quality, arm position at injury, vector of force, and pre-existing tendon degeneration. Detection and quantification of partial-thickness and full-thickness tears remain important because management depends on tear depth, size, symptoms, and functional impairment [18,19].

The representative MRI images in Figures 4-6 demonstrate the practical imaging spectrum in this study. Figure 4 shows supraspinatus tendinosis, an important finding because tendinosis may coexist with trauma and should not be overcalled as a tear. Figure 5 shows a full-thickness U-shaped supraspinatus tear with fluid signal traversing the tendon and medial retraction. Figure 6 shows Ellman Grade I and Grade III partial-thickness supraspinatus tears. These examples align with recent attempts to identify MRI features that distinguish traumatic from degenerative cuff pathology [20].

From a clinical perspective, early MRI characterization may influence patient triage. Asymptomatic tears can progress and become symptomatic over time [21], and traumatic events may convert a compensated shoulder into a symptomatic one. Comprehensive reviews of rotator cuff disease have emphasized that management depends on symptoms, tear size, tissue quality, age, activity level, and functional demand [22]. In this context, mechanism-based MRI interpretation can help identify patients who may benefit from early orthopedic referral, particularly those with full-thickness tears, larger tears, multi-tendon involvement, tendon retraction, and bone marrow edema.

Partial-thickness tears require careful description because they may be treated conservatively, arthroscopically debrided, or repaired depending on depth and symptoms. The management principles for partial-thickness tears have emphasized that higher-grade lesions are more clinically significant than low-grade surface fraying [23]. In the present study, Ellman Grade III tears represented a smaller but important subgroup because these deep partial-thickness defects approach full-thickness involvement and may influence management decisions.

Associated findings also deserve mention. Joint effusion, subacromial-subdeltoid bursitis, long head of biceps tendon pathology, labral tear, and fractures were observed in varying proportions. Historically, shoulder arthrography and MR arthrography have been used to evaluate cuff and intra-articular pathology [24,25]. ABER-position MR arthrography has also been described for improving the assessment of anterior glenoid labral tears [26]. Although the present study used conventional non-arthrographic MRI, fat-saturated multiplanar sequences provided clinically useful information for traumatic cuff assessment.

The high frequencies of supraspinatus involvement, tendon edema, joint effusion, and associated fractures should be interpreted in the context of the study’s eligibility criteria and referral pathway. The cohort did not represent an unselected population with shoulder trauma; rather, it included patients referred for MRI because of clinical suspicion of trauma-induced rotator cuff injury and required MRI confirmation of a cuff abnormality for inclusion. This selection process may have enriched the study population for more conspicuous tendon abnormalities and associated traumatic findings, including osseous injuries. Accordingly, these frequencies describe the characteristics of the present cohort and should not be interpreted as population-level prevalence estimates for all patients with traumatic shoulder injury.

Potential pitfalls must also be considered. Tendinosis, magic-angle artifact, small interstitial tears, and normal anatomic variants may mimic pathology. MRI interpretation of the shoulder requires awareness of such refinements to avoid overdiagnosis or underdiagnosis [27]. In the present study, structured evaluation helped separate tendinosis-only cases from partial-thickness and full-thickness tear components, improving the clarity of results.

Rehabilitation and functional recovery depend not only on tendon anatomy but also on coordinated muscle activity around the shoulder. Shoulder muscle function during rehabilitation has been studied extensively, emphasizing the role of balanced strengthening and controlled loading [28]. Therefore, MRI findings should be interpreted in a clinical framework that includes pain, strength, function, age, activity level, and rehabilitation potential. Imaging severity does not always exactly match symptoms, but it provides an anatomical basis for planning treatment.

The findings should be interpreted with limitations. This was a single-institution study with a modest sample size. Surgical or arthroscopic confirmation was not available in all patients. Chronic microscopic degeneration could not be completely excluded, particularly in older patients. Some subgroup comparisons had small numbers and zero-cell distributions, making Fisher’s exact test more appropriate in selected analyses. The prevalence and risk factors of rotator cuff tears vary across populations [29], and subscapularis tears may require dedicated imaging attention for accurate detection [30]. Additional limitations include potential observer bias because, consistent with the mechanism-based study design, the readers were not blinded to the documented clinical history or mechanism of injury during MRI interpretation. Interobserver agreement was also not assessed. Selection bias may also have been introduced because the study included only patients referred for MRI with clinical suspicion of rotator cuff injury, rather than an unselected population with shoulder trauma. Furthermore, multivariable analysis was not performed because of the modest sample size and small subgroup counts, limiting adjustment for potential confounders such as age, pre-existing tendon degeneration, injury severity, and associated osseous trauma. Accordingly, the observed mechanism-based relationships should be interpreted as unadjusted associations rather than independent predictive effects. Therefore, larger multicenter studies with surgical correlation and follow-up outcomes are needed.

Despite these limitations, the study identified several clinically relevant MRI patterns in trauma-induced rotator cuff injuries. The principal findings were universal supraspinatus involvement, frequent multi-tendon injury, predominance of partial-thickness tears, and significant associations between the mechanism of injury and tear type, Cofield tear-size category, and bone marrow edema. Integrating the clinical mechanism with tear morphology, acute injury markers, and associated shoulder findings may improve the interpretation of traumatic rotator cuff injuries and provide useful information for subsequent clinical management.

## Conclusions

MRI provides a comprehensive and clinically useful assessment of trauma-induced rotator cuff injuries by defining tendon involvement, tear type, tear depth, full-thickness tear size, tendon retraction, acute injury markers, and associated intra-articular or periarticular abnormalities. In this study, supraspinatus involvement was the most consistent finding, while multi-tendon involvement was frequent. Partial-thickness tears were the predominant injury pattern, either in isolation or in combination with full-thickness tear components.

Mechanism-based analysis demonstrated significant associations between the nature of trauma and the MRI pattern of injury. High-energy trauma was predominantly associated with full-thickness tear components, larger supraspinatus tears, and bone marrow edema, whereas lower-energy mechanisms more commonly demonstrated partial-thickness tear patterns. These findings support a structured MRI approach that integrates the mechanism of injury with tear morphology, acute injury markers, and associated shoulder abnormalities. Such correlation may assist diagnostic interpretation, help differentiate acute traumatic injury from degenerative or acute-on-chronic pathology, and provide clinically relevant information for subsequent orthopedic assessment and management planning.
